# Ovary Activation Dynamics in the Bean Weevil *Zabrotes subfasciatus* (Bruchinae): The Essential Roles of Seeds and Males

**DOI:** 10.3390/insects16090894

**Published:** 2025-08-27

**Authors:** Sílvia de Oliveira Miranda, Bruno de Oliveira Cruz, Juliana Ramos Martins, Talita Sarah Mazzoni, Waner de Oliveira Miranda, Lívia Maria Rosatto Moda, Ester Siqueira Caixeta, Isabel Ribeiro do Valle Teixeira, Angel Roberto Barchuk

**Affiliations:** 1 Departamento de Biologia Celular e do Desenvolvimento, Instituto de Ciências Biomédicas, Universidade Federal de Alfenas, UNIFAL-MG, Rua Gabriel Monteiro Silva, 700, Alfenas 37130-001, MG, Brazil; silvia.miranda@sou.unifal-mg.edu.br (S.d.O.M.); bruno.cruz@sou.unifal-mg.edu.br (B.d.O.C.); juliana.ramos@unifal-mg.edu.br (J.R.M.); talita.mazzoni@unifal-mg.edu.br (T.S.M.); livia.rosatto@unifal-mg.edu.br (L.M.R.M.); ester.caixeta@unifal-mg.edu.br (E.S.C.); 2 Microsoft, São Paulo 04543-907, SP, Brazil; wanermiranda@gmail.com; 3 Microsoft, Redmond, WA 98052, USA; 4 Instituto Federal Sul de Minas-IFSULDEMINAS-Campus, Poços de Caldas 37713-100, MG, Brazil; 5 Laboratório de Biologia Molecular-M-206, Centro de Biologia Experimental-CEBIOEX, Universidade Federal de Alfenas, UNIFAL-MG, Campus Santa Clara, Av. Jovino Fernandes Sales, 2.600, Alfenas 37133-840, MG, Brazil

**Keywords:** ovary activation, vitellogenesis, oviposition, vitellogenin, juvenile hormone, host seed, VOCs, bean weevil, *Zabrotes subfasciatus*, Bruchinae

## Abstract

Phytophagous beetles, particularly those within the superfamilies Chrysomeloidea and Curculionoidea, constitute one of the most diverse and ecologically significant groups of insect herbivores. Within this group, the subfamily Bruchinae is especially notable for its close association with leguminous plant seeds. As most Bruchinae species do not feed during the adult stage, the timing and regulation of vitellogenesis remain unclear. Previous studies suggest that vitellogenesis may be triggered by volatile organic compounds emitted by host seeds, which promote juvenile hormone (JH) synthesis. This increase in JH is hypothesized to stimulate vitellogenesis, enhance female attractiveness, and ultimately facilitate fertilization and oviposition. To explore this hypothesis, we investigated the external cues regulating reproductive physiology in the capital breeder *Zabrotes subfasciatus*. Specifically, we examined the effects of host seeds and male presence on oviposition dynamics, fecundity, ovary activation, and the expression of vitellogenic genes (*vg* and *vgR*) throughout adult life. Our results show that females initiate vitellogenesis during the final phases of adult development, enabling oviposition to begin as early as the first day after emergence. Oviposition remains at basal levels throughout adult life unless both host seeds and males are present (*p* < 0.0001). This oviposition pattern is consistent with ovary activation dynamics, which reveal that vitellogenesis peaks early in the oviposition period and is prolonged by the presence of seeds and males (*p* < 0.05). Notably, *vg* and *vgR* gene expression respond differentially to these cues (*p* < 0.05). We integrate our findings with previous literature to propose a working model for the regulation of oviposition in the Bruchinae beetle *Z. subfasciatus*.

## Simple Summary

Here we address the poorly understood regulation of vitellogenesis—the production of egg yolk proteins—in adult female Bruchinae beetles, which do not feed during adulthood. Focusing on *Zabrotes subfasciatus*, a seed-feeding beetle, we aimed to determine how environmental cues such as host seed presence and male interaction influence reproductive processes. By analyzing oviposition timing, fecundity, ovary activation, and expression of vitellogenic genes (*vg* and *vgR*), we found that vitellogenesis begins during late adult development, allowing oviposition to start on the first day post-emergence. However, sustained and elevated egg-laying requires both seeds and male presence, which also prolong vitellogenic activity and enhance ovary activation. The differential gene expression in response to these cues supports the role of external stimuli in regulating reproduction. We conclude with a proposed model linking environmental signals to hormonal and genetic mechanisms governing oviposition. These findings deepen our understanding of insect reproductive strategies, particularly in capital breeders, and could inform pest management strategies by identifying key triggers in beetle reproduction, ultimately contributing to improved agricultural sustainability.

## 1. Introduction

Phytophagous beetles, particularly those from the superfamilies Chrysomeloidea and Curculionoidea, along with lepidopterans and hemipterans, constitute the majority of insects that feed on plants. With approximately 135,000 species [1], these insects have achieved significant biological success, utilizing seeds, leaves, trunks, and wood as their primary food sources. This success is largely attributed to the acquisition of genes encoding enzymes capable of degrading plant cell wall compounds, which were obtained through horizontal gene transfer from bacteria and fungi [2,3].

The family Chrysomelidae, specifically the subfamily Bruchinae (with approximately 1700 species) [4], is particularly notable for its association with plants in the Fabaceae (Leguminosae) family [5,6]. The association between Bruchinae beetles and dry legume seeds appears to be ancestral, likely favoured by long periods of drought and the availability of stored seeds [7,8]. In these insects, eggs are deposited onto the pods or directly onto the seeds. Upon hatching, the larvae burrow into the walls of the pods and/or seeds, feeding on the interior of the seeds as they grow and develop until reaching the pupal stage (which lasts between 7 and 28 days). The larvae then emerge as adults, mate, and complete a new cycle. In some species, the pupal development occurs outside the seeds, with the cocoon either attached to the seeds or directly to the soil [6]. Most adult beetles, particularly those of greater economic concern due to their infestation of stored seeds, do not feed. However, some species have the ability to consume food during their adult life [9], and in those inhabiting more “natural” niches, food intake seems to enhance reproductive capacity [6].

As the eggs deposited on seeds give rise to immatures that develop within them, members of this subfamily are considered pests [8]. Infestations by these insects lead to significant losses, with up to a 20% reduction in the value of grains during pre-processing and packaging [10,11,12]. However, paradoxically, despite the economic importance of beetles that prey on legume seeds, and aside from ecological and evolutionary studies that use some reproductive parameters [13,14,15,16], the developmental biology of Bruchinae species remains poorly understood. *Zabrotes subfasciatus*, for instance, has been used to explore molecular and physiological aspects of digestion [17,18] and the adaptation of phytophagous insects to new hosts [19,20,21]. With the availability of its genomic sequence (Accession: PRJNA1192986), *Z. subfasciatus* has the potential to become a key biological model for molecular studies in Bruchinae. This could be beneficial both from an experimental manipulation standpoint—its natural niche is easily replicable in the lab—and from a representational perspective, as insights gained from studying *Z. subfasciatus* could inform the understanding and management of other Bruchinae species [22]. Additionally, using *Z. subfasciatus* as a model offers several advantages, such as the ease of distinguishing individuals by sex (females and males) and the ability to identify seeds that have been oviposited upon, whether fertilized or not, allowing for precise and efficient analysis [23,24].

*Z. subfasciatus* Boheman (1833) [25] (Coleoptera; Chrysomeloidea; Chrysomelidae; Bruchinae) [26] is a beetle native to Central and South America, from where it has spread worldwide, primarily to tropical regions that cultivate Fabaceae [24,27]. This species can prey on a variety of Fabaceae, but it prefers varieties of common beans (*Phaseolus vulgaris*), where it demonstrates comparatively high fitness [22,28,29,30]. As with other Bruchinae species, females deposit and attach their eggs to the surface of a host seed. After the embryo develops, the larva hatches, penetrates the seed coat, and feeds on the cotyledons and embryonic regions of the seed. After completing pupal development, the adults emerge—typically after 26 days of development at 30–35 °C [9]—undergo a brief 10-day reproductive period, and then die [21].

The predominant mechanism by which insects attempt to optimize their reproductive capacity in response to environmental conditions is likely the modulation of oocyte maturation rates [31]. In Orthoptera, Blattodea, Hemiptera, and Coleoptera, where reproduction depends on adult nutrition or mating, vitellogenesis is strictly regulated by juvenile hormone (JH) [32,33,34]. Many different external stimuli (mediated by hormones, as JH) are known to modulate oocyte maturation rates in various insects: temperature, humidity, food availability, social regulation, etc. [31]. In *Z. subfasciatus*, as in other studied Bruchinae species (e.g., *Acanthoscelides obtectus*, *Bruchidius atrolineatus*, *Callosobruchus maculatus*, and *Caryedon serratus*), ovarian development is facilitated only when the host plants are at, or nearly at, the phenological stage suitable for first-instar larvae to feed [12,35,36,37,38]. Behavioural assays suggest that males are attracted to females for mating, but only after the females are exposed to oviposition substrates [38,39,40], which increase ovary development [41] so that females are capable of ovipositing starting from the first day of adulthood [21].

Considering *Z. subfasciatus* adults do not usually feed, it is unclear whether vitellogenesis occurs during post-embryonic development (e.g., in the later phases of the pupal period), or whether its induction in adults depends on the presence of a male (and subsequent mating), similar to what occurs in *Drosophila* and *Anastrepha* [42,43,44,45]. Alternatively, does vitellogenesis depend on the presence of oviposition substrates, such as Fabaceae seeds, as seen in other beetles like the burying beetle (*Nicrophorus orbicollis*), or is it a combination of both factors, as suggested by Pimbert and Pierre [41]? In *N. orbicollis* (Polyphaga, Silphidae), which does not feed on the carcass where it lays eggs, JH levels increase dramatically within 20 min of contact with the substrate, inducing vitellogenesis, which then occurs in the following 18 h [46,47]. If vitellogenesis in *Z. subfasciatus* depends on a factor derived from seeds (a chemosensory factor), the events must also be rapid, simultaneously promoting vitellogenesis, enhancing female attractiveness, and allowing fertilization. A system that could facilitate these events might involve volatile organic compounds (VOCs) emitted by seeds [20] and/or the involvement of maxillary palps [48], JH production to promote vitellogenesis, and pheromone release to attract males.

In this study, we aimed to elucidate how external environmental and social cues regulate the reproductive physiology of the seed beetle *Z. subfasciatus*. Our specific objectives were to (1) investigate the influence of the developmental substrate (i.e., host seeds) on female oviposition patterns, fecundity, and reproductive timing; (2) evaluate the impact of male presence on reproductive output throughout the adult lifespan; (3) examine ovary activation dynamics under varying experimental conditions; and (4) assess the expression profiles of key vitellogenic genes (vitellogenin and vitellogenin receptor) involved in reproductive maturation and egg production [49]. Finally, we integrated these findings to propose a working model describing the regulation of reproductive physiology in *Z. subfasciatus*, highlighting the interaction between environmental cues and internal reproductive processes.

## 2. Materials and Methods

### 2.1. Beetle Husbandry

The *Z. subfasciatus* individuals used in this study were derived from a stock population of approximately 6000 individuals, initially collected from bean seeds in Ribeirão Preto, São Paulo, Brazil, and maintained on bean seeds in the laboratory since 1997. According to Souza et al. [50], Brazilian populations of *Z. subfasciatus* exhibit low genetic differentiation and weak geographic structure. To mitigate potential inbreeding effects, individuals collected from bean seeds in Poços de Caldas and Alfenas, Minas Gerais, Brazil, have been incorporated into the stock population approximately every three months since 2007. The stock population was maintained on bean seeds at 22 ± 5 °C and 70 ± 15% relative humidity, under dark conditions. Additional details regarding the biology of this insect can be found in many studies [24,51,52,53].

### 2.2. Histological Processing of Pupal and Adult Ovaries

To determine whether *Z. subfasciatus* females emerge with activated ovaries or if this activation occurs only in newly emerged individuals, we collected beetles maintained on beans from the stock. Ovaries dissected under a stereomicroscope (Stemi 305, Carl Zeiss, Jena, Germany) from adult and pupae stage individuals were fixed in Karnovsky’s solution [54] at 4 °C for 48 h. Both the pupae and the adult ovaries were dehydrated in a series of ethanol concentrations: 70%, 80%, 90%, and 95% (2 changes of 30 min each at room temperature). They were then gradually embedded in resin (Historesin embedding kit, Leica Biosystems, Nussloch, Germany), starting with a 2:1 (*v*/*v*) ratio of 95% ethanol to resin, followed by ratios of 1:1 and 1:2 (1 h each at room temperature). Finally, the specimens were incubated in pure resin for 48 h at 4 °C. Blocks were prepared according to the kit’s instructions. Sections were made using a Leica RM2135 rotary microtome (Leica Biosystems, Nussloch, Germany), at a thickness of 5 μm, with high-profile blades (818, Leica Biosystems, Nussloch, Germany). The material was stained with Harris’ hematoxylin and eosin (H&E; Synth^®^, Labsynth, Diadema-SP, Brazil) [55]. Micrographs were captured using an Olympus microscope (Olympus Corporation, Hachioji, Tokyo, Japan), equipped with a digital camera (AmScope-MU1003, Irvine, CA, USA) and AmLite imaging software (version 1.0, AmScope, Irvine, CA, USA).

### 2.3. Experimental Group Allocation: Groups A, B, C, and D

Virgin females were selected from individual infested bean seeds, identified by the presence of darkened, unbroken opercula. These seeds were placed in 10 mL test tubes sealed with cotton and maintained under controlled environmental conditions of 70% humidity and 30 °C in a BOD incubator (EL101/03, Eletrolab, São Paulo, SP, Brazil), considered optimal for species development [28,30,56].

After emergence, individuals were distributed into acrylic tubes (3.5 cm in height, 2 cm at the base, and 3 cm in upper diameter), sealed with fine-mesh tulle fabric secured with an elastic band, and allocated into four experimental groups: only females (Group A), females with males but without host seeds (Group B), females with host seeds but without males (Group C), and females with both males and host seeds (Group D). The experiment was set up with 18 replicates per experimental group. The tubes from groups C and D included 10 bean seeds to ensure enough oviposition substrate thus avoiding competition [16,57].

#### 2.3.1. Assessment of the Oviposition and Fecundity Profiles

To ensure accurate daily oviposition records, the tubes were replaced every 24 h with new ones containing an equal number of host seeds. Ten days after the final replacement-when the eggs became visible-egg counts were conducted on both the inner surfaces of the tubes and the host seeds. In the groups where insect emergence was possible (B and D), adult emergence was recorded between days 25 and 26 following the last replacement. These data were used to estimate fecundity under the experimental conditions.

#### 2.3.2. Dissection of Adult Ovaries, Fluorescent Staining, and Scoring of Ovariole Activation

To assess the impact of host seed availability and mate presence on *Z. subfasciatus* ovary activation, newly emerged females (0–24 h old), as well as females aged 4 and 8 days from each experimental group (10 replicates per day per group), were dissected under a stereomicroscope (Stemi 305, Carl Zeiss, Jena, Germany) to obtain ovaries. Ovaries were dissected in 1× phosphate-buffered saline (PBS; Sigma Aldrich, St. Louis, MO, USA) and fixed for 24 h at 4 °C in 200 µL of n-heptane (Vetec-920178, Rio de Janeiro, Brazil) and 37% formaldehyde (Proquímicos-10/0010, São Paulo, Brazil).

After fixation, ovaries were washed twice (5 min each) in PBST, a solution containing PBT and 0.5% Triton X-100 (Sigma-Aldrich, St. Louis, MO, USA) and incubated at room temperature for 20 min in a solution of phalloidin conjugated to Alexa 546 (1:500 *v*/*v* ratio in PBST; Invitrogen, Carlsbad, CA, USA) to stain F-actin, primarily highlighting cytoplasmic structures. Subsequently, samples were washed three times (5 min each) in PBST and incubated for 4 min in a DAPI solution (Sigma-Aldrich, St. Louis, MO, USA) at a 1:2000 *v*/*v* ratio in PBST. After a final wash (three times, 10 min each) in PBST, ovaries were mounted in 80% glycerol (Sigma-Aldrich, St. Louis, MO, USA) and examined under a fluorescence microscope (Eclipse 80i, Nikon, Tokyo, Japan) and a confocal microscope C2+ (Nikon, Tokyo, Japan). Ovariole activation was assessed based on the degree of vitellogenesis in the oocytes, and females were classified into two stages: Stage I, comprising individuals with primarily early-stage oocytes, and Stage II, comprising those with predominantly mature, yolk-rich oocytes.

#### 2.3.3. Quantification of Vitellogenic Genes Transcript Levels Using RT-qPCR

Total RNA was extracted using TRIzol^®^ (Ambion, Thermo Fisher Scientific, Waltham, MA, USA) from pools (4 pools each) of three carcasses (body without head, ovaries, ovipositor, and intestine) and five pairs of ovaries and processed as previously reported [20]. Briefly, to eliminate contaminant DNA, total RNA was incubated at 37 °C with 1 unit of RQ1 RNase-free DNase (Promega, Madison, WI, USA) for 30 min, followed by enzyme inactivation at 65 °C for 10 min using 1 µL of Stop DNase Solution (Promega, Madison, WI, USA). First-strand cDNA was synthesized from 1 μg of total RNA via reverse transcription using SuperScript^®^ IV Reverse Transcriptase (Invitrogen, Carlsbad, CA, USA) and Oligo dT12-18 primers (Invitrogen, Carlsbad, CA, USA). Negative control reactions, lacking the enzyme, were conducted in parallel. To quantitatively compare gene transcript levels between populations, a real-time qPCR assay was performed using a QuantStudio 3 PCR System (Applied Biosystems, Foster City, CA, USA). Amplifications were carried out in 10 μL reaction volumes, each containing 5 μL SYBR Green Master Mix 2X (Applied Biosystems, Foster City, CA, USA), 1 μL first-strand cDNA (diluted 1:4 *v*/*v* in water), 3.2 μL nuclease-free water (Thermo Fisher Scientific, Waltham, MA, USA), and 0.4 μL of each specific primer (from a 10 μM stock solution; Table S1). The gene encoding *Z. subfasciatus* ribosomal protein L32 (*rpl32*) was used as the reference gene [20]. Negative controls included reactions without reverse transcriptase or template. PCR conditions were set as follows: 50 °C for 2 min, 95 °C for 10 min, followed by 40 cycles of 95 °C for 15 s and 60 °C for 1 min. To ensure reproducibility, each SYBR Green assay was conducted in technical duplicates, with at least three biological replicates [58], from the four pools per tissue, as mentioned above.

### 2.4. Data Analysis

Statistical analyses began with the Shapiro–Wilk test to assess the normality of all datasets. Since the total number of eggs laid across treatments followed a normal distribution, comparisons among groups were conducted using one-way ANOVA, followed by Tukey’s multiple comparisons test to identify significant differences. To examine the number of emerged adults over time in the group of females with males and host seeds (Group D), a two-way repeated measures ANOVA was applied, as the same individuals were monitored across multiple time points. Šídák’s multiple comparisons test was used to assess differences between days. On day 2 of the experiment, differences among the four experimental groups were analyzed using the Kruskal–Wallis’s test followed by Dunn’s multiple comparisons test. This non-parametric approach was chosen due to the non-normal distribution of the data at this specific time point. To evaluate the level of ovarian activation (stages I and II) under different experimental conditions, categorical data (presence or absence of oocyte stages) were analyzed using Fisher’s exact test.

For the quantification of vitellogenic gene transcript levels by RT-qPCR, raw data were normalized to the reference gene *rpl32*, and relative transcript quantities were calculated using the 2^−ΔΔCt^ method with efficiency correction and a calibrator sample [20,59] (Applied Biosystems User Bulletin #2). Since these data did not follow a normal distribution, the Kruskal–Wallis’s test was again employed, followed by Dunn’s multiple comparisons test. However, for comparisons between pupae and the other experimental groups, the Mann–Whitney U test was applied, as the pupa group had a small sample size and was not included in the main experimental design. All statistical analyses and graph construction were performed using GraphPad Prism version 10.5.0 (GraphPad Software, Boston, MA, USA, www.graphpad.com, accessed on 17 July 2025) [60]. Differences were considered statistically significant when *p* ≤ 0.05.

## 3. Results

### 3.1. Female Zabrotes subfasciatus Begin Vitellogenesis During the Final Phases of Adult Development

**Figure 1:**
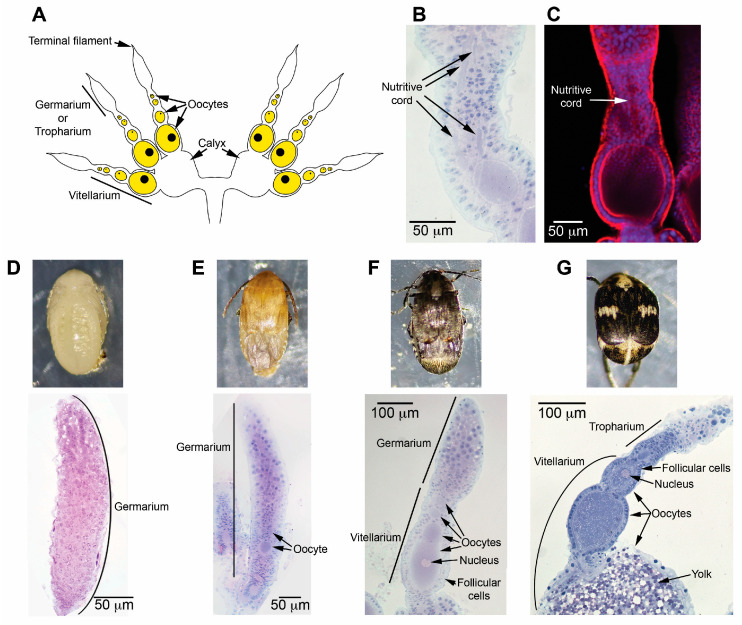
Ovary activation in *Zabrotes subfasciatus* occurs during the pharate-adult stage, prior to adult emergence. The ovaries (except that in (**C**)) were embedded in Historesin, sectioned to a thickness of 5 μm, and stained with Harris’ hematoxylin and eosin [55]. Micrographs were captured using a Nikon Eclipse 80i microscope equipped with a digital camera and Nis-Element 3.1 imaging software. For the image in (**C**), ovary was processed for 4′,6-diamidino-2-phenylindole (DAPI) and phalloidin conjugated to Alexa 546 and the micrograph captured in a confocal microscope C2^+^ Nikon. (**A**) The diagram illustrates the ovary of an adult insect. (**B**,**C**) Histological and confocal images display a telotrophic ovariole highlighting nutritive cords. (**D**–**G**): Representative images of females and ovariole sections; (**D**) white-eyed pupa; (**E**,**F**) final pharate-adult phase; (**G**) one-day-old adult female.

Histological analysis of newly emerged *Z. subfasciatus* ovaries confirmed the presence of three ovarioles per ovary, exhibiting a telotrophic meroistic organization (Figure 1A–C). Additionally, examination of ovarian activation revealed that vitellogenesis is initiated during the final phases of adult development (Figure S1 and Figure 1). During the larval phases and the early phase of pupal development—specifically in white-eyed pupae—the ovarioles consist solely of a mass of germ and somatic cells with no signs of oocyte development (Figure S1 and Figure 1D). In early adult development ovarioles contain immature oocytes, with no visible signs of vitellogenin uptake (Figure 1E). As development advances, oocytes begin to accumulate yolk in defined regions (Figure 1F), progressively resembling the fully developed oocytes observed in newly emerged females (Figure 1G).

### 3.2. Oviposition in Zabrotes subfasciatus Begins Shortly After Adult Emergence and Is Enhanced by the Availability of Seeds and Males

**Figure 2:**
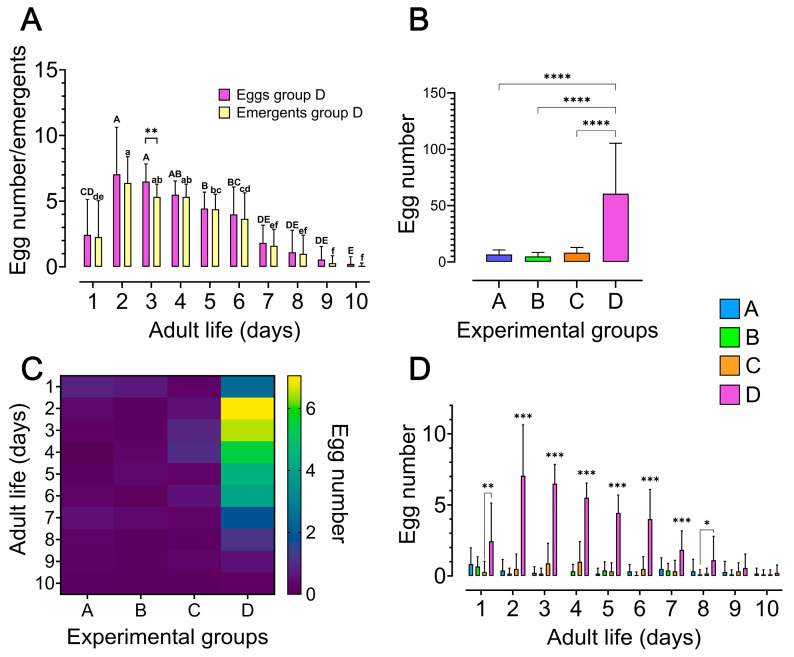
Impact of host seed availability and mate presence on oviposition behaviour and fecundity of *Zabrotes subfasciatus* females. Eighteen newly emerged females per experimental group were individually placed in acrylic containers. Group A: females only; Group B: females and males; Group C: females and seeds; Group D: females, males, and seeds. The number of laid eggs was registered daily as well as the number of adults emerged from each of the laid egg during the 10 days of the oviposition period. (**A**) Oviposition and adult emergence profiles of the females from experimental group D: The number of emerged adults represents the individuals that developed from eggs laid on each specific day during the oviposition period. Different uppercase letters on the bars indicate statistically significant differences in the mean number of eggs laid during the oviposition period and different lowercase letters indicate statistically significant differences in the mean number of emergents (comparing the means of the different days). Two-way repeated measures ANOVA, followed by Šídák’s multiple comparisons test, ** *p* < 0.01. (**B**) Total number of eggs laid per female during the oviposition period (mean + SD). Ordinary one-way ANOVA followed Tukey’s multiple comparisons test, **** *p* < 0.0001. (**C**) Heatmap depicting the mean number of eggs laid per female in each of the 10-day oviposition period. (**D**) Oviposition profile per female during the 10-day oviposition period (mean + SD). * *p* < 0.05; ** *p* < 0.01; *** *p* < 0.001. For statistical details, see Tables S2–S5.

In ideal conditions (with seeds and males), *Z. subfasciatus* females began ovipositing on the first day after emergence, reaching a peak on the second day with 7 ± 3.5 eggs (mean ± SD; Kruskal–Wallis’s test followed by Dunn’s multiple comparisons test *p* < 0.0001). After this peak, oviposition gradually declined until day 10 (Figure 2A,B; Tables S2–S5). The number of eggs laid generally corresponded to the number of emerging individuals, except on day 3, when a significant difference was observed (Figure 2A; two-way repeated measures ANOVA, followed by Šídák’s multiple comparisons test *p* < 0.05; Tables S3 and S4). Females without access to an oviposition substrate and/or males laid a total of 3 ± 0.2 eggs (mean ± SD) throughout their lifespan, whereas those provided with both substrate and males laid significantly more (60 ± 44 eggs mean ± SD; ordinary one-way ANOVA followed by Tukey’s multiple comparisons test *p* < 0.0001; Figure 2B; Table S5). This pattern was further validated by daily oviposition records. Notably, only females from experimental group D (with both oviposition substrate and males) exhibited significantly higher oviposition rates compared to all other groups (on days 1 and 8 the difference was observed only in relation to the females of groups C and B, respectively; Figure 2C,D; Table S5). Females in group C exhibited a slight, though non-significant, increase in egg-laying on days 3 and 4 compared to those in groups A and B (Figure 2D).

### 3.3. Vitellogenesis Is Elevated During the Early Phase of the Oviposition Period in Zabrotes subfasciatus, and Its Duration Is Extended by the Presence of Seeds and Males

**Figure 3:**
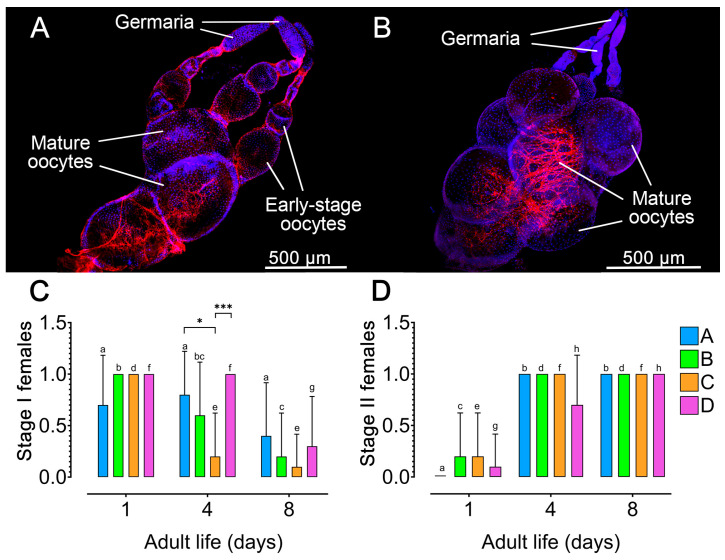
Impact of host seed availability and mate presence on the ovary activation of adult *Zabrotes subfasciatus* females. Eighteen newly emerged females per experimental group were individually placed in acrylic containers. Group A: females only; Group B: females and males; Group C: females and seeds; Group D: females, males, and seeds. The upper images depict representative photographs of ovarioles with oocytes in different levels of development (**A**), (Stage I ovarioles) and an ovary with fully developed oocytes (**B**), (Stage II ovarioles). Ten females from each experimental group were dissected and the activation level of their ovarioles was registered as corresponding to Stage I (**C**) or Stage II (**D**). Different letters on the columns with values from the same experimental group (colour) over the time studied indicate statistically significant differences (*p* < 0.05). Data are presented as mean + SD. Fisher’s exact test, * *p* < 0.01 and *** *p* < 0.001. For statistical details, see Tables S6–S9.

To investigate how the experimental environment—specifically host and male presence—affects the reproductive physiology of *Z. subfasciatus*, we assessed ovarian activation in females from the four experimental groups (Figure 3A,B; Tables S6 and S7). Across all groups, a consistent pattern emerged: females were predominantly in Stage I at the beginning of the oviposition period (notably on day 1), and in Stage II by the end of the period (day 8), indicating a significant temporal shift in ovarian development (Fisher’s exact test, *p* < 0.05; Figure 3C,D; Tables S8 and S9). Moreover, by day 4, group D exhibited a higher proportion of females in Stage I and a lower proportion in Stage II compared to the other groups (Figure 3C,D). However, the difference was statistically significant only between the mean of groups D and C regarding females in Stage I (Fisher’s exact test *p* = 0.0007). The mean values between females from the groups A and C regarding Stage I were also statistically different (Fisher’s exact test *p* = 0.023).

### 3.4. Male Presence Enhances Vitellogenin (Vg) Expression, While Seeds Boost Vitellogenin Receptor (vgR) Expression in the Ovaries of Zabrotes subfasciatus Females

**Figure 4:**
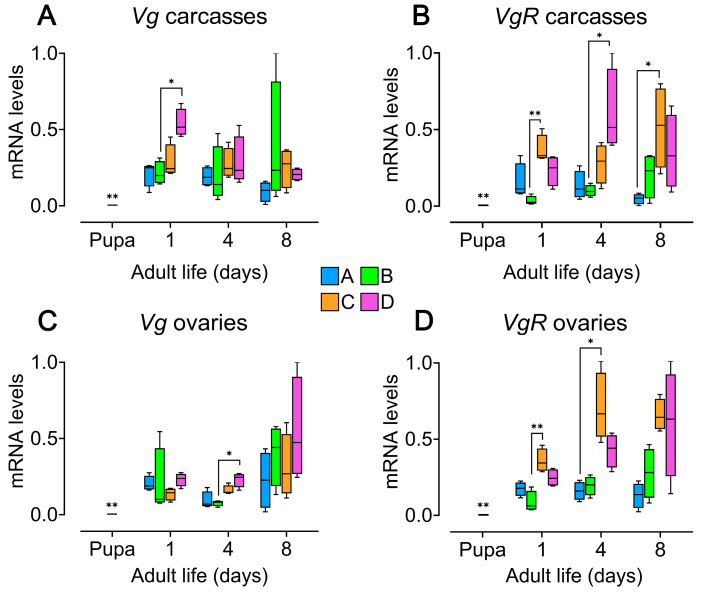
Impact of host seed availability and mate presence on the expression of vitellogenic genes in *Zabrotes subfasciatus* females. Eighteen newly emerged females per experimental group were individually placed in acrylic containers. Group A: females only; Group B: females and males; Group C: females and seeds; Group D: females, males, and seeds. Tissues from adult females were dissected and 4 RNA pools from 5 individuals were subjected to RT-qPCR (whole body of pupae were processed). Raw data were first normalized to the reference gene (rpl32) and the relative quantities of transcripts were calculated using the 2^−ΔΔCt^ method with efficiency correction and a control sample for calibration [59] (Applied Biosystems User bulletin #2). Y axes: 2^−ΔΔCt^ values. Comparisons with the pupal stage, as well as between experimental groups and days, were performed using the Mann–Whitney and test Kruskal–Wallis’s test followed by Dunn’s multiple comparisons test. Median and interquartile range (25th–75th percentiles), with minimum and maximum values; * *p* < 0.05; ** *p* < 0.01. (**A**) *Vg* gene transcript levels in carcass tissues, which include fat body. (**B**) *VgR* gene transcript levels in carcass tissues. (**C**) *Vg* gene transcript levels in ovary tissues. (**D**) *VgR* gene transcript levels in ovary tissues. For statistical details, see Tables S10–S17.

All analyzed tissues from adult *Z. subfasciatus* females exhibited higher levels of vitellogenic genes [61] transcription compared to pharate-adult females, in which transcription was nearly undetectable. Both tested genes (*vg* and *vgR*) were expressed in the carcass and ovaries across all female age groups and experimental conditions (Figure 4; Tables S10–S14). Median levels of *vg* gene transcripts in the carcass were generally consistent among all experimental groups throughout the study period, except on day 1. On this day, females from group D showed significantly higher *vg* expression than those from the other groups, with a statistically significant difference observed compared to group B (Figure 4A; Kruskal–Wallis’s test followed by Dunn’s multiple comparisons test, *p* < 0.05; Table S15). Expression of the *vgR* gene in carcass tissue showed more variability. On day 1, females from group C had significantly higher *vgR* transcript levels than those in group B, and on day 8, they exceeded those in group A (Figure 4B; Kruskal–Wallis’s test followed by Dunn’s multiple comparisons test, *p* < 0.05; *p* < 0.01; Table S15). Additionally, group D females showed significantly greater *vgR* expression than group B on day 4 (Figure 4B; Kruskal–Wallis’s test followed by Dunn’s multiple comparisons test, *p* < 0.05; Table S15). In ovarian tissues, *vg* transcript levels were consistently higher in group D females, though the difference reached statistical significance only on day 4 compared to group B (Figure 4C; Kruskal–Wallis’s test followed by Dunn’s multiple comparisons test, *p* < 0.05; Table S16). Lastly, *vgR* expression also varied among groups. The highest median expression levels were consistently observed in females from group C, with statistically significant differences compared to group B on day 1 and group A on day 4 (Figure 4D; Kruskal–Wallis’s test followed by Dunn’s multiple comparisons test, *p* < 0.05; *p* < 0.01; Table S16).

## 4. Discussion

We first confirmed that adults *Z. subfasciatus* carry three ovarioles per ovary, each with telotrophic meroistic organization, and showed that vitellogenesis initiates during the final phases of adult development. Under ideal conditions, females began ovipositing on the first day after emergence, reaching a peak on the second day, after which the oviposition gradually declines until day 10. Oviposition is at basal low levels during the whole adult life of the females unless both seeds and males are present. The observed oviposition profile was further confirmed by the ovary activation (vitellin accumulation) dynamics which revealed vitellogenesis is elevated during the early phase of the oviposition period and its duration is extended by the presence of seeds and males, which affects differentially vitellogenin and vitellogenin receptor expression.

Both ovariole number and type are genetically determined, with ovariole type further reflecting phylogenetic relationships. Some of the ovary characteristics, though may suffer alterations from cellular perturbations during the later phases of larval development and can be modified by the diet of the immature stages [62]. As reported previously for other Bruchinae [63,64,65], females *Z. subfasciatus* have ovaries with three telotrophic meroistic ovarioles. Each telotrophic ovariole has an anterior tropharium, in which nurse cells remain, synthesize and contribute their products finally into all growing oocytes via nutritive cords [66,67].

As predicted by the concept of capital breeding [68], based on reports on the nutritional biology of Bruchinae [6], we showed that *Z. subfasciatus* begin vitellogenesis during the final phases of adult development. Since there is no food intake during the adult development, the result suggests that during the larval feeding period, females accumulate nutrients in their fat body cells, which contributes to the development of oocytes during later post-embryonic phases and early days of adult life.

Our results show that oviposition, as a result of ovary activation and vitellogenesis, concentrates during the early days after adult emergence, with a peak already in the second day of adult life, thus partially agreeing with the finding reported in Teixeira et al. [21]. These authors showed that the beetles reared on beans have an oviposition maximum already on the first day after emergence. However, the authors also showed that variations in the host seed types may shift the observed oviposition peak.

Females *Z. subfasciatus* show basal low levels of oviposition during the whole adult life unless both seeds (host) and males are present. This result indicates that both factors are essential for promoting oviposition levels compatible with populational maintenance and growth, as suggested in an early work by Pimbert and Pierre [41]. Our results also show that the high levels of oviposition registered during the first days of adult life are fuelled by high levels of oocyte development, as evidenced by the high proportion of females in the Stage I of ovary activation, including developing and fully developed oocytes. This condition was registered to last during the following days in the females reared in the presence of seed and males. During the last part of the adult life, however, the ovaries of females from all experimental groups are in Stage II, i.e., mainly with fully developed oocytes. Interestingly, the females reared in the ideal condition showed the lowest proportion of fully developed oocytes retained within their reproductive system, at least before reaching the final days of the adult life. This result suggests that vitellogenesis might not depend directly on external factors and egg laying (evidenced by the proportionally low level of retained fully developed oocytes) is enhanced by the presence of males in females with available host seeds (the latter also suggested by Pimbert and Pierre [41]). Moreover, the observation that females from all experimental groups retain Stage II ovaries until the end of their lives is noteworthy and warrants further investigation. This may indicate an inability to lay eggs, potentially linked to ageing processes in females, males, or even to social factors. The latter is particularly plausible given that, in natural settings, females can encounter other females and may mate with multiple males [69]—conditions that could influence oviposition behaviour [70].

Vitellogenin has been shown to be a pleiotropic protein, performing multiple functions beyond its traditional role in yolk-protein formation in insects [71]. In line with this, we found that the vitellogenin gene—and that of its receptor, *vgR*—is expressed in all analyzed tissues, including both the carcass and ovary. The expression of *vgR* in the carcass appears to be regulated in a complex manner by external factors, also warranting further investigation. This pattern of widespread expression is consistent with findings in the bumblebee [72], and notably includes ovarian expression, as also observed in the Chagas disease vector *Rhodnius prolixus* [73]. Interestingly, although *vg* is expressed at very low levels in pharate adults, its presence at this stage aligns with the detection of vitellogenic oocytes, suggesting an early onset of reproductive activity. These findings collectively indicate that basal *vg* transcription and its incorporation into oocytes are regulated by internal physiological signals—likely hormonal—and that the production of these cues may be further amplified by external environmental factors.

The expression of *vg* in the carcass (which includes the fat body) and ovaries is enhanced -at least at certain points during the oviposition period- by the presence of males when host seeds are available. In contrast, *vgR* expression in the ovaries appears to depend primarily on the availability of an oviposition substrate, suggesting that *vg* expression in the fat body and *vg* uptake by oocytes are physiological processes regulated by distinct external cues. As reported in other insect species, *vg* gene expression can be induced by JH [33]. *vgR*, however, has been described as being expressed in a relatively autonomous manner in some insects [74,75], a pattern that also appears to apply to the basal expression levels observed in *Z. subfasciatus*. Similar to findings in the beet armyworm *Spodoptera exigua* [76], *vgR* expression in the bean beetle is strongly modulated by the presence of an oviposition substrate- in this case, host seeds.

## 5. Conclusions

**Figure 5:**
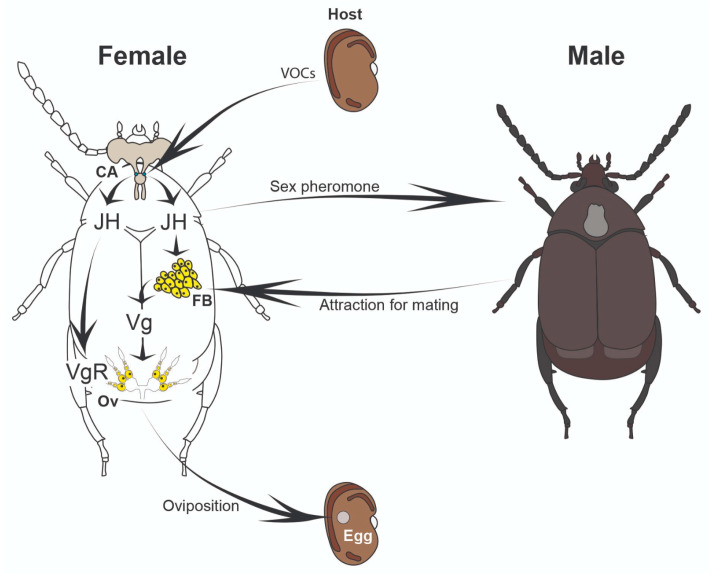
Proposed working model of oviposition regulation in the seed beetle *Zabrotes subfasciatus*. The proposed increase in juvenile hormone (JH) synthesis following female exposure to host seeds, as well as the chemical nature of the sex pheromone involved, require experimental validation. CA = corpora allata; FB = fat body; Ov = ovaries; JH = juvenile hormone; Vg = vitellogenin; VgR = vitellogenin receptor.

Our results indicate that the capital breeder *Z. subfasciatus* initiates vitellogenesis during the final phases of adult development. This process is likely fuelled by nutrients stored in the fat body cells during the larval stage. Consequently, the female’s physiological system is primed to begin oviposition shortly after adult emergence. Based on our findings and previous literature, we propose a model in which host seeds emit VOCs [77] that enhance JH synthesis in females [38,40]. Elevated JH levels, in turn, trigger the production and release of sex pheromones [40,41,78], promoting male attraction and facilitating mating. JH also induces the expression of the vitellogenin receptor (*vgR*) in the ovaries (and potentially *vg* in fat bodies), while the increased mating activity further modulates vitellogenin (*vg*) expression and promotes oviposition (Figure 5).

## Data Availability

The original contributions presented in this study are included in the article/Supplementary Material. Further inquiries can be directed to the corresponding author.

## Supplementary Materials

The following supporting information can be downloaded at: https://www.mdpi.com/article/10.3390/insects16090894/s1, Table S1: Characteristics of the primers used in the qPCR assays; Table S2: Descriptive statistics of daily oviposition in Groups A (females only), B (females and males), and C (females and seeds), and adult emergence (EM) in Group D (females, males and seeds) over a 10-day oviposition period; Table S3: Comparison of oviposition and adult emergence in Group D (females, males and seeds) across 10 days using two-way RM ANOVA with Šídák’s test; Table S4: Statistical comparison of daily oviposition in Group D (females, males and seeds) across the 10-day period using Kruskal–Wallis test followed by Dunn’s multiple comparisons test; Table S5: Statistical comparison of total oviposition among experimental groups: A (females only), B (females and males), C (females and seeds), and D (females, males and seeds), using one-way ANOVA followed by Tukey’s multiple comparisons test; Table S6: Descriptive statistics of Stage I ovarioles in Groups A (females only), B (females and males), C (females and seeds), and D (females, males and seeds). Females were dissected at 1, 4, and 8 days of age. Representative images of Stage I ovarioles are shown in figure 3- panel A; Table S7: Descriptive statistics of Stage II ovarioles in Groups A (females only), B (females and males), C (females and seeds), and D (females, males and seeds). Females were dissected at 1, 4, and 8 days of age. Representative images of Stage II ovarioles are shown in figure 3- panel B; Table S8: Statistical comparison of the number of females with Stage I and Stage II ovaries across days 1, 4, and 8 within each experimental group: A (females only), B (females and males), C (females and seeds), and D (females, males, and seeds), using Fisher’s exact test; Table S9: Statistical comparison of the number of females with Stage I and Stage II ovaries among experimental groups A (females only), B (females and males), C (females and seeds), and D (females, males, and seeds), at days 1, 4, and 8, using Fisher’s exact test; Table S10: Descriptive statistics of normalized *VgR* transcript levels (2^^−ΔΔCt^ values) in carcass tissues of females from Groups A (females only), B (females and males), C (females and seeds), and D (females, males and seeds). Tissues were obtained from adult females, and four RNA pools (n = 5 individuals each) were analyzed by RT-qPCR. Relative expression was calculated using the 2^^−ΔΔCt^ method with efficiency correction and normalization to rpl32; Table S11: Descriptive statistics of normalized *Vg* transcript levels (2^^−ΔΔCt^ values) in carcass tissues of females from Groups A (females only), B (females and males), C (females and seeds), and D (females, males and seeds). Tissues were obtained from adult females, and four RNA pools (n = 5 individuals each) were analyzed by RT-qPCR. Relative expression was calculated using the 2^^−ΔΔCt^ method with efficiency correction and normalization to rpl32; Table S12: Descriptive statistics of normalized *Vgr* transcript levels (2^^−ΔΔCt^ values) in ovarie tissues of females from Groups A (females only), B (females and males), C (females and seeds), and D (females, males and seeds). Tissues were obtained from adult females, and four RNA pools (n = 5 individuals each) were analyzed by RT-qPCR. Relative expression was calculated using the 2^–ΔΔCt method with efficiency correction and normalization to rpl32; Table S13: Descriptive statistics of normalized *Vg* transcript levels (2^^−ΔΔCt^ values) in ovarie tissues of females from Groups A (females only), B (females and males), C (females and seeds), and D (females, males and seeds). Tissues were obtained from adult females, and four RNA pools (n = 5 individuals each) were analyzed by RT-qPCR. Relative expression was calculated using the 2^^−ΔΔCt^ method with efficiency correction and normalization to rpl32; Table S14: Normalized *Vg* and *VgR* transcript levels (2^^−ΔΔCt^ values) in carcass and ovaries of pupae: descriptive statistics from RT-qPCR data (3 pools, n = 5) normalized to rpl32; Table S15: Statistical comparison of *Vg* and *VgR* transcript levels (2^^−ΔΔCt^ values) in carcasses tissues among experimental groups A (females only), B (females and males), C (females and seeds), and D (females, males, and seeds), within each time point (days 1, 4, and 8), using Kruskal–Wallis test followed by Dunn’s multiple comparisons test; Table S16: Statistical comparison of *Vg* and *VgR* transcript levels (2^^−ΔΔCt^ values) in ovaries tissues among experimental groups A (females only), B (females and males), C (females and seeds), and D (females, males, and seeds), within each time point (days 1, 4, and 8), using Kruskal–Wallis test followed by Dunn’s multiple comparisons test; Table S17: Statistical comparison of *Vg* and *VgR* transcript levels (2^^−ΔΔCt^ values) in carcass and ovarian tissues of pupae among experimental groups A (females only), B (females and males), C (females and seeds), and D (females, males, and seeds), using Mann-Whitney test; Figure S1: Ovariole activation during the development of *Zabrotes subfasciatus.*
