# Supplementary material for: Ovary Activation Dynamics in the Bean Weevil Zabrotes subfasciatus (Bruchinae): The Essential Roles of Seeds and Males
Source: Insects. 2025 Aug 27;16(9):894. doi: 10.3390/insects16090894 (PMC12470986; doi:10.3390/insects16090894)
Supplement: Supplementary file 1 [file insects-16-00894-s001.zip › insects-3796791-supplementary.pdf]

Ovary activation dynamics in the bean weevil *Zabrotes subfasciatus* (Bruchinae): the essential roles of seeds and males

Sílvia de Oliveira Miranda[1], Bruno de Oliveira Cruz[1], Juliana Ramos Martins[1], Talita Sarah Mazzoni[1], Waner de Oliveira Miranda[2], Lívia Maria Rosatto Moda[1], Ester Siqueira Caixeta Nogueira[1], Isabel Ribeiro do Valle Teixeira[3], Angel Roberto Barchuk[1*]

[1]Departamento de Biologia Celular e do Desenvolvimento, Instituto de Ciências Biomédicas, Universidade Federal de Alfenas, UNIFAL-MG, Alfenas, Minas Gerais, Brazil.
[2]Senior Software Engineer, Microsoft, São Paulo, Brazil; Redmond, Washington, USA.
[3]Instituto Federal Sul de Minas – IFSULDEMINAS - Campus Poços de Caldas, Minas Gerais, Brazil.

Supplementary Material
Tables and Figure

Table S1. Characteristics of the primers used in the qPCR assays. Amplification temperature = 60 °C. Vg.g1.i1 = vitellogenin gene 1, isoform 1. VgR.g1.i1 = vitellogenin receptor gene 1, isoform 1. Rpl32 = ribosomal protein 32 [20].

Gene	Primer Sequence		Amplicon (bp)
	Forward	Reverse	
Vg.g1.i1	CCATCCAGAACTGGATTCTC	ATGAACGAGCTCGGTGTATG	197
VgR.g1.i1	GGATACCGATGGACACGTTT	TTCTGACTACGGTTGGTGTG	137
rpl32	TACACAACGTGAGGGAACTG	AGCATGTCCGTTCGTTACTC	146

Table S2. Descriptive statistics of daily oviposition in Groups A (females only), B (females and males), and C (females and seeds), and adult emergence (EM) in Group D (females, males and seeds) over a 10-day oviposition period. Number of values (N), Mean, Standard deviation (SD), Standard error of mean (SEM), Median and Sum calculated in Graphpad Prism software (10.5.0).

Group	N	Day	Mean	SD	SEM	Median	Sum
A	18	1	0.8333	1.15	0.2712	0	15
		2	0.3889	0.7775	0.1833	0	7
		3	0.2222	0.4278	0.1008	0	4
		4	0	0	0	0	0
		5	0.1667	0.3835	0.09039	0	3
		6	0.3333	0.4851	0.1143	0	6
		7	0.5	0.7859	0.1852	0	9
		8	0.3333	0.8402	0.198	0	6
		9	0.2778	0.7519	0.1772	0	5
		10	0.1667	0.3835	0.09039	0	3
B	18	1	0.6667	0.686	0.1617	1	12
		2	0.1667	0.3835	0.09039	0	3
		3	0.1667	0.3835	0.09039	0	3
		4	0.3333	0.4851	0.1143	0	6
		5	0.3889	0.6077	0.1432	0	7
		6	0.05556	0.2357	0.05556	0	1
		7	0.3889	0.5016	0.1182	0	7
		8	0.1111	0.3234	0.07622	0	2
		9	0.1111	0.3234	0.07622	0	2
		10	0.1111	0.3234	0.07622	0	2
C	18	1	0.2778	0.7519	0.1772	0	5
		2	0.5	1.043	0.2459	0	9
		3	0.8889	1.41	0.3322	0	16
		4	1	1.414	0.3333	1	18
		5	0.3333	0.5941	0.14	0	6
		6	0.5	0.8575	0.2021	0	9
		7	0.3333	0.767	0.1808	0	6
		8	0.1667	0.3835	0.09039	0	3
		9	0.3333	0.5941	0.14	0	6
		10	0.1111	0.3234	0.07622	0	2
D	18	1	2.444	2.684	0.6326	1.5	44
		2	7.056	3.572	0.842	6.5	127
		3	6.5	1.339	0.3157	6	117
		4	5.5	1.043	0.2459	5	99
		5	4.444	1.247	0.294	4.5	80
		6	4	2.086	0.4918	4	72

		**7**	1.833	1.339	0.3157	1.5	33
		**8**	1.111	1.676	0.3951	0.5	20
		**9**	0.5556	0.9835	0.2318	0	10
		**10**	0.2222	0.5483	0.1292	0	4
**EM**	**18**	**1**	2.278	2.761	0.6508	1	41
		**2**	6.389	2.004	0.4724	6	115
		**3**	5.333	0.9701	0.2287	5	96
		**4**	5.333	0.9701	0.2287	5	96
		**5**	4.389	1.145	0.2698	4.5	79
		**6**	3.667	1.97	0.4644	4	66
		**7**	1.611	1.243	0.293	1.5	29
		**8**	1	1.414	0.3333	0	18
		**9**	0.2778	0.5745	0.1354	0	5
		**10**	0.05556	0.2357	0.05556	0	1

Table S3. Comparison of oviposition and adult emergence in Group D (females, males and seeds) across 10 days using two-way RM ANOVA with Šídák's test. Mean difference (Mean Diff.) and p-value calculated in Graphpad Prism software (10.5.0).

Šídák's multiple comparisons test (day)	Mean diff.	Adjusted P Value
1	0.1667	>0.9999
2	0.6667	0.9535
3	1.167	0.0135
4	0.1667	0.9821
5	0.05556	>0.9999
6	0.3333	0.9779
7	0.2222	0.9821
8	0.1111	0.9989
9	0.2778	0.4379
10	0.1667	0.8741

Table S4. Statistical comparison of daily oviposition in Group D (females, males and seeds) across the 10-day period using Kruskal–Wallis test followed by Dunn's multiple comparisons test. Mean rank difference (Mean rank Diff.) and p-value calculated in Graphpad Prism software (10.5.0).

Dunn's multiple comparisons test	Mean rank diff.	Adjusted P Value
day 1 vs. day 2	-72.47	0.0011
day 1 vs. day 3	-76.92	0.0003
day 1 vs. day 4	-60.39	0.0192
day 1 vs. day 5	-40.25	0.8497
day 1 vs. day 6	-31.39	>0.9999
day 1 vs. day 7	4.917	>0.9999
day 1 vs. day8	22.36	>0.9999
day 1 vs. day9	35.36	>0.9999
day 1 vs. day 10	42.39	0.6035
day 2 vs. day 3	-4.444	>0.9999
day 2 vs. day 4	12.08	>0.9999
day 2 vs. day 5	32.22	>0.9999
day 2 vs. day 6	41.08	0.7449
day 2 vs. day7	77.39	0.0003
day 2 vs. day 8	94.83	<0.0001
day 2 vs. day 9	107.8	<0.0001
day 2 vs. day 10	114.9	<0.0001
day 3 vs. day 4	16.53	>0.9999
day 3 vs. day 5	36.67	>0.9999
day 3 vs. day 6	45.53	0.3561
day 3 vs. day 7	81.83	<0.0001
day 3 vs. day 8	99.28	<0.0001
day 3 vs. day 9	112.3	<0.0001
day 3 vs. day 10	119.3	<0.0001
day 4 vs. day 5	20.14	>0.9999
day 4 vs. day 6	29	>0.9999
day 4 vs. day 7	65.31	0.0063
day 4 vs. day 8	82.75	<0.0001
day 4 vs. day 9	95.75	<0.0001
day 4 vs. day 10	102.8	<0.0001
day 5 vs. day 6	8.861	>0.9999
day 5 vs. day 7	45.17	0.379
day 5 vs. day 8	62.61	0.0117
day 5 vs. day 9	75.61	0.0005
day 5 vs. day 10	82.64	<0.0001
day 6 vs. day 7	36.31	>0.9999
day 6 vs. day 8	53.75	0.0772
day 6 vs. day 9	66.75	0.0044
day 6 vs. day 10	73.78	0.0008
day 7 vs. day 8	17.44	>0.9999
day 7 vs. day 9	30.44	>0.9999

day 7 vs. day 10	37.47	>0.9999
day 8 vs. day 9	13	>0.9999
day 8 vs. day 10	20.03	>0.9999
day 9 vs. day 10	7.028	>0.9999

Table S5. Statistical comparison of total oviposition among experimental groups: A (females only), B (females and males), C (females and seeds), and D (females, males and seeds), using one-way ANOVA followed by Tukey's multiple comparisons test. Mean difference (Mean Diff.) and p-value calculated in Graphpad Prism software (10.5.0).

Tukey's multiple comparisons test (group)	Mean diff.	Adjusted P Value
A vs. B	1.6	0.9985
A vs. C	-1.7	0.9982
A vs. D	-54	<0.0001
B vs. C	-3.3	0.987
B vs. D	-55.6	<0.0001
C vs. D	-52.3	<0.0001

Table S6. Descriptive statistics of Stage I ovarioles in Groups A (females only), B (females and males), C (females and seeds), and D (females, males and seeds). Females were dissected at 1, 4, and 8 days of age. Representative images of Stage I ovarioles are shown in figure 3- panel A. Number of values (N), Mean, Standard deviation (SD), Standard error of mean (SEM) and Frequency calculated in Graphpad Prism software (10.5.0).

Group	Day	N	Mean	SD	SEM	Frequency
**A**	**1**	10	0.7	0.483046	0.1528	7
	**4**	10	0.8	0.421637	0.1333	8
	**8**	10	0.4	0.516398	0.1633	4
**B**	**1**	10	1	0	0	10
	**4**	10	0.6	0.5164	0.1633	6
	**8**	10	0.2	0.4216	0.1333	2
**C**	**1**	10	1	0	0	10
	**4**	10	0.2	0.4216	0.1333	2
	**8**	10	0.1	0.3162	0.1	1
**D**	**1**	10	1	0	0	10
	**4**	10	1	0	0	10
	**8**	10	0.3	0.483	0.1528	3

Table S7. Descriptive statistics of Stage II ovarioles in Groups A (females only), B (females and males), C (females and seeds), and D (females, males and seeds). Females were dissected at 1, 4, and 8 days of age. Representative images of Stage II ovarioles are shown in figure 3- panel B. Number of values (N), Mean, Standard deviation (SD), Standard error of mean (SEM) and Frequency calculated in Graphpad Prism software (10.5.0).

Group	Day	N	Mean	SD	SEM	Frequency
**A**	**1**	10	0	0	0	0
	**4**	10	1	0	0	10
	**8**	10	1	0	0	10
**B**	**1**	10	0.2	0.4216	0.1333	2
	**4**	10	1	0	0	10
	**8**	10	1	0	0	10
**C**	**1**	10	0.2	0.4216	0.1333	2
	**4**	10	1	0	0	10
	**8**	10	1	0	0	10
**D**	**1**	10	0.1	0.3162	0,1	1
	**4**	10	0.7	0.483	0.1528	7
	**8**	10	1	0	0	10

Table S8. Statistical comparison of the number of females with Stage I and Stage II ovaries across days 1, 4, and 8 within each experimental group: A (females only), B (females and males), C (females and seeds), and D (females, males, and seeds), using Fisher's exact test. P-value calculated in Graphpad Prism software (10.5.0).

Stage	Group	Fisher's exact test (day)	P Value
I	A	1 vs. 4	>0.9999
		1 vs. 8	0.3698
		4 vs. 8	0.1698
	B	1 vs. 4	0.0867
		1 vs. 8	0.0007
		4 vs. 8	0.1698
	C	1 vs. 4	0.0007
		1 vs. 8	0.0001
		4 vs. 8	>0.9999
	D	1 vs. 4	>0.9999
		1 vs. 8	0.0031
		4 vs. 8	0.0031
II	A	1 vs. 4	<0.0001
		1 vs. 8	<0.0001
		4 vs. 8	>0.9999
	B	1 vs. 4	0.0007
		1 vs. 8	0.0007
		4 vs. 8	>0.9999
	C	1 vs. 4	0.0007
		1 vs. 8	0.0007
		4 vs. 8	>0.9999
	D	1 vs. 4	0.0198
		1 vs. 8	0.0001
		4 vs. 8	0.2105

Table S9. Statistical comparison of the number of females with Stage I and Stage II ovaries among experimental groups A (females only), B (females and males), C (females and seeds), and D (females, males, and seeds), at days 1, 4, and 8, using Fisher's exact test. P-value calculated in Graphpad Prism software (10.5.0).

Stage	Fisher's exact test (group)	Day 1	Day 4	Day 8
		P Value	P Value	P Value
I	**A x B**	0.2105	0.6285	0.6285
	**A x C**	0.2105	0.023	0.3034
	**A x D**	0.2105	0.4137	>0.9999
	**B x C**	>0.9999	0.1698	>0.9999
	**B x D**	>0.9999	0.0867	>0.9999
	**C x D**	>0.9999	0.0007	0.582
II	**A x B**	0.4737	>0.9999	>0.9999
	**A x C**	0.4737	>0.9999	>0.9999
	**A x D**	>0.9999	>0.9999	>0.9999
	**B x C**	>0.9999	>0.9999	>0.9999
	**B x D**	>0.9999	0.2105	>0.9999
	**C x D**	>0.9999	0.2105	>0.9999

Table S10. Descriptive statistics of normalized *VgR* transcript levels (2^–ΔΔCt values) in carcass tissues of females from Groups A (females only), B (females and males), C (females and seeds), and D (females, males and seeds). Tissues were obtained from adult females, and four RNA pools (n = 5 individuals each) were analyzed by RT-qPCR. Relative expression was calculated using the 2^–ΔΔCt method with efficiency correction and normalization to rpl32. Number of values (N), Mean, Standard deviation (SD), Standard error of mean (SEM) and Sum calculated in Graphpad Prism software (10.5.0).

Group	Day	N (pools)	Mean	SD	SEM	Median	Sum
A	**1**	4	0.1575	0.1162	0.05811	0.1111	0.63
	**4**	4	0.1321	0.09303	0.04652	0.1105	0.5284
	**8**	4	0.04717	0.03402	0.01701	0.05189	0.1887
B	**1**	4	0.03526	0.0295	0.01475	0.0246	0.141
	**4**	4	0.09862	0.03775	0.01888	0.09419	0.3945
	**8**	4	0.2021	0.148	0.07398	0.2305	0.8082
C	**1**	4	0.3686	0.09203	0.04602	0.3286	1.474
	**4**	4	0.2783	0.1295	0.06474	0.2932	1.113
	**8**	4	0.5157	0.2696	0.1348	0.5278	2.063
D	**1**	4	0.2325	0.1007	0.05033	0.2495	0.9298
	**4**	4	0.6059	0.2738	0.1369	0.5135	2.424
	**8**	4	0.3499	0.243	0.1215	0.3279	1.4

Table S11. Descriptive statistics of normalized *Vg* transcript levels (2^–ΔΔCt values) in carcass tissues of females from Groups A (females only), B (females and males), C (females and seeds), and D (females, males and seeds). Tissues were obtained from adult females, and four RNA pools (n = 5 individuals each) were analyzed by RT-qPCR. Relative expression was calculated using the 2^–ΔΔCt method with efficiency correction and normalization to rpl32. Number of values (N), Mean, Standard deviation (SD), Standard error of mean (SEM) and Sum calculated in Graphpad Prism software (10.5.0).

Group	Day	N (pools)	Mean	SD	SEM	Median	Sum
**A**	**1**	4	0.2117	0.08392	0.04196	0.2486	0.8467
	**4**	4	0.1904	0.06416	0.03208	0.1855	0.7617
	**8**	4	0.09215	0.06601	0.03301	0.1003	0.3686
**B**	**1**	4	0.2119	0.07673	0.03836	0.1965	0.8478
	**4**	4	0.1971	0.1886	0.09432	0.1381	0.7883
	**8**	4	0.3813	0.4209	0.2105	0.2324	1.525
**C**	**1**	4	0.2867	0.1103	0.05515	0.2434	1.147
	**4**	4	0.2723	0.1006	0.0503	0.244	1.089
	**8**	4	0.2497	0.1306	0.06531	0.2749	0.9987
**D**	**1**	4	0.5383	0.09312	0.04656	0.5149	2.153
	**4**	4	0.2859	0.1646	0.0823	0.2323	1.144
	**8**	4	0.2043	0.03992	0.01996	0.2037	0.8173

Table S12. Descriptive statistics of normalized *Vgr* transcript levels (2^–ΔΔCt values) in ovarie tissues of females from Groups A (females only), B (females and males), C (females and seeds), and D (females, males and seeds). Tissues were obtained from adult females, and four RNA pools (n = 5 individuals each) were analyzed by RT-qPCR. Relative expression was calculated using the 2^–ΔΔCt method with efficiency correction and normalization to rpl32. Number of values (N), Mean, Standard deviation (SD), Standard error of mean (SEM) and Sum calculated in Graphpad Prism software (10.5.0).

Group	Day	N (pools)	Mean	SD	SEM	Median	Sum
A	**1**	4	0.1723	0.04492	0.02246	0.1761	0.6893
	**4**	4	0.1588	0.05837	0.02919	0.1587	0.6353
	**8**	4	0.13	0.08185	0.04092	0.1356	0.5199
B	**1**	4	0.08728	0.06825	0.03412	0.06351	0.3491
	**4**	4	0.1933	0.06233	0.03116	0.1982	0.7733
	**8**	4	0.2743	0.1624	0.08118	0.2785	1.097
C	**1**	4	0.3548	0.0754	0.0377	0.3405	1.419
	**4**	4	0.6972	0.2217	0.1109	0.6582	2.789
	**8**	4	0.6501	0.102	0.05101	0.6349	2.601
D	**1**	4	0.245	0.05638	0.02819	0.2421	0.9798
	**4**	4	0.4226	0.1086	0.05432	0.4357	1.69
	**8**	4	0.5974	0.3523	0.1761	0.6237	2.389

Table S13. Descriptive statistics of normalized *Vg* transcript levels (2^–ΔΔCt values) in ovarie tissues of females from Groups A (females only), B (females and males), C (females and seeds), and D (females, males and seeds). Tissues were obtained from adult females, and four RNA pools (n = 5 individuals each) were analyzed by RT-qPCR. Relative expression was calculated using the 2^–ΔΔCt method with efficiency correction and normalization to rpl32. Number of values (N), Mean, Standard deviation (SD), Standard error of mean (SEM) and Sum calculated in Graphpad Prism software (10.5.0).

Group	Day	N (pools)	Mean	SD	SEM	Median	Sum
**A**	**1**	4	0.2021	0.05071	0.02535	0.1867	0.8084
	**4**	4	0.09177	0.05747	0.02874	0.06884	0.3671
	**8**	4	0.2248	0.1871	0.09353	0.2251	0.8991
**B**	**1**	4	0.2046	0.2271	0.1135	0.09931	0.8185
	**4**	4	0.07516	0.01853	0.009264	0.08114	0.3006
	**8**	4	0.3969	0.2015	0.1008	0.4395	1.588
**C**	**1**	4	0.1346	0.04105	0.02052	0.1427	0.5385
	**4**	4	0.1588	0.03168	0.01584	0.1453	0.6353
	**8**	4	0.3119	0.2103	0.1051	0.2685	1.248
**D**	**1**	4	0.23	0.04433	0.02217	0.2373	0.9198
	**4**	4	0.2296	0.04855	0.02427	0.2446	0.9184
	**8**	4	0.5471	0.3402	0.1701	0.4719	2.189

Table S14. Normalized *Vg* and *VgR* transcript levels (2^−ΔΔCt values) in carcass and ovaries of pupae: descriptive statistics from RT-qPCR data (3 pools, n = 5) normalized to rpl32. Number of values (N), Mean, Standard deviation (SD), Standard error of mean (SEM) and Sum calculated in Graphpad Prism software (10.5.0).

	N (pools)	Mean	SD	SEM	Median	Sum
**Ovaries *Vg***	3	0.0001297	0.0001003	0.00005792	0.000181	0.0003891
**Ovaries *Vgr***	3	0.005404	0.004418	0.002551	0.003701	0.01621
**Carcasses *Vg***	3	0.000001675	0.000001208	0.0000006976	0.00000136	0.000005026
**Carcasses *Vgr***	3	0.000008587	0.000003719	0.000002147	0.0000072	0.00002576

Table S15. Statistical comparison of *Vg* and *VgR* transcript levels (2^–ΔΔCt values) in carcasses tissues among experimental groups A (females only), B (females and males), C (females and seeds), and D (females, males, and seeds), within each time point (days 1, 4, and 8), using Kruskal–Wallis test followed by Dunn's multiple comparisons test. Mean rank difference (Mean rank Diff.) and p-value calculated in Graphpad Prism software (10.5.0).

Tissue	Day	Dunn's multiple comparisons test (group)	Mean rank diff.	Adjusted P Value
Carcasses *Vgr*	1	A vs. B	5.75	0.5258
		A vs. C	-5.25	0.7133
		A vs. D	-1.5	>0.9999
		B vs. C	-11	0.0065
		B vs. D	-7.25	0.1876
		C vs. D	3.75	>0.9999
	4	A vs. B	1	>0.9999
		A vs. C	-4.25	>0.9999
		A vs. D	-8.75	0.0561
		B vs. C	-5.25	0.7133
		B vs. D	-9.75	0.0227
		C vs. D	-4.5	>0.9999
	8	A vs. B	-4.25	>0.9999
		A vs. C	-9.5	0.0286
		A vs. D	-7.25	0.1876
		B vs. C	-5.25	0.7133
		B vs. D	-3	>0.9999
		C vs. D	2.25	>0.9999
Carcasses *Vg*	1	A vs. B	1	>0.9999
		A vs. C	-1	>0.9999
		A vs. D	-8	0.1049
		B vs. C	-2	>0.9999
		B vs. D	-9	0.0451
		C vs. D	-7	0.2255
	4	A vs. B	0.75	>0.9999
		A vs. C	-3.75	>0.9999
		A vs. D	-4	>0.9999
		B vs. C	-4.5	>0.9999
		B vs. D	-4.75	0.9495
		C vs. D	-0.25	>0.9999
	8	A vs. B	-6.25	0.3803
		A vs. C	-7	0.2255
		A vs. D	-5.75	0.5258
		B vs. C	-0.75	>0.9999
		B vs. D	0.5	>0.9999
		C vs. D	1.25	>0.9999

Table S16. Statistical comparison of *Vg* and *VgR* transcript levels (2^–ΔΔCt values) in ovaries tissues among experimental groups A (females only), B (females and males), C (females and seeds), and D (females, males, and seeds), within each time point (days 1, 4, and 8), using Kruskal–Wallis test followed by Dunn's multiple comparisons test. Mean rank difference (Mean rank Diff.) and p-value calculated in Graphpad Prism software (10.5.0).

Tissue	Day	Dunn's multiple comparisons test (group)	Mean rank diff.	Adjusted P Value
Ovaries Vgr	1	A vs. B	3.5	>0.9999
		A vs. C	-7.75	0.128
		A vs. D	-3.75	>0.9999
		B vs. C	-11.25	0.005
		B vs. D	-7.25	0.1876
		C vs. D	4	>0.9999
	4	A vs. B	-1.5	>0.9999
		A vs. C	-10.25	0.014
		A vs. D	-7.25	0.1876
		B vs. C	-8.75	0.0561
		B vs. D	-5.75	0.5258
		C vs. D	3	>0.9999
	8	A vs. B	-2.25	>0.9999
		A vs. C	-8.75	0.0561
		A vs. D	-7	0.2255
		B vs. C	-6.5	0.3211
		B vs. D	-4.75	0.9495
		C vs. D	1.75	>0.9999
Ovaries Vg	1	A vs. B	4.25	>0.9999
		A vs. C	4.5	>0.9999
		A vs. D	-1.75	>0.9999
		B vs. C	0.25	>0.9999
		B vs. D	-6	0.4482
		C vs. D	-6.25	0.3803
	4	A vs. B	0.5	>0.9999
		A vs. C	-4.75	0.9477
		A vs. D	-8.75	0.0558
		B vs. C	-5.25	0.7117
		B vs. D	-9.25	0.0358
		C vs. D	-4	>0.9999
	8	A vs. B	-4	>0.9999
		A vs. C	-1.25	>0.9999
		A vs. D	-5.75	0.5258
		B vs. C	2.75	>0.9999
		B vs. D	-1.75	>0.9999
		C vs. D	-4.5	>0.9999

Table S17. Statistical comparison of *Vg* and *VgR* transcript levels (2^–ΔΔCt values) in carcass and ovarian tissues of pupae among experimental groups A (females only), B (females and males), C (females and seeds), and D (females, males, and seeds), using Mann-Whitney test. Direct difference of medians (Diff.), Mann-Whitney U (U) and p-value calculated in Graphpad Prism software (10.5.0).

Tissue	Mann Whitney test	Diff.	U	P value
Carcasses *Vg*	Pupa vs. A	0.1507	0	0.0022
	Pupa vs. B	0.1855	0	0.0022
	Pupa vs. C	0.2434	0	0.0022
	Pupa vs. D	0.2405	0	0.0022
Carcasses *Vgr*	Pupa vs. A	0.0839	3	0.0154
	Pupa vs. B	0.08032	0	0.0022
	Pupa vs. C	0.3372	0	0.0022
	Pupa vs. D	0.3552	0	0.0022
Ovaries *Vg*	Pupa vs. A	0.1681	0	0.0022
	Pupa vs. B	0.09913	0	0.0022
	Pupa vs. C	0.1535	0	0.0022
	Pupa vs. D	0.248	0	0.0022
Ovaries *Vgr*	Pupa vs. A	0.1523	0	0.0022
	Pupa vs. B	0.1817	0	0.0022
	Pupa vs. C	0.57	0	0.0022
	Pupa vs. D	0.3455	0	0.0022

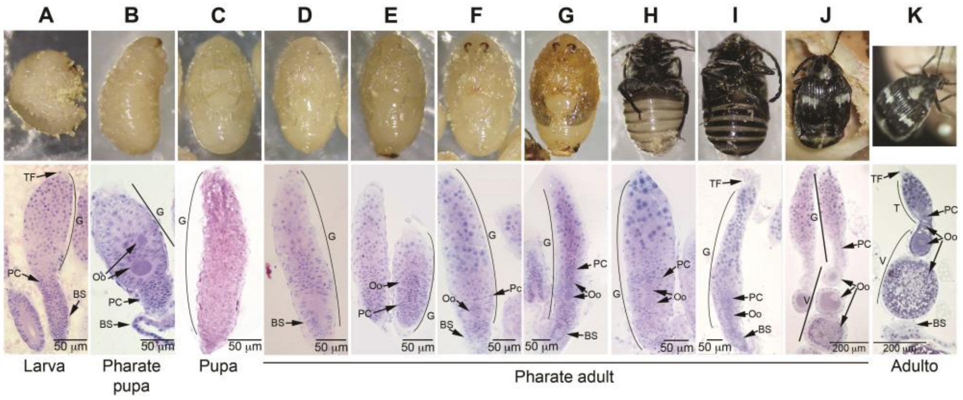

Figure S1. Ovariole activation during the development of *Zabrotes subfasciatus*. Representative photographs of A) larva; B) pharate pupa; C) pupa; D – J) pharate adults, and K) adult insects along with their respective histological images of ovarioles. The ovaries were embedded in Historesin, sectioned to a thickness of 5 µm, and stained with Harris' hematoxylin and eosin [55]. Micrographs were captured using a Nikon Eclipse 80i microscope equipped with a digital camera and Nis-Element 3.1 imaging software.
